# Current concepts on ventricular fibrillation: A Vicious Circle of Cardiomyocyte Calcium Overload in the Initiation, Maintenance, and Termination of Ventricular Fibrillation

**Published:** 2004-04-01

**Authors:** Christian E Zaugg

**Affiliations:** Department of Research, Experimental Cardiology Research Group, University Hospital Basel, Switzerland

**Keywords:** calcium overload, ventricular fibrillation, defibrillation, myocardial stunning

## Abstract

Based on recent experimental studies, this review article introduces the novel concept that  cardiomyocyte Ca^2+^ and ventricular fibrillation (VF) are mutually related, forming a self-maintaining  vicious circle in the initiation, maintenance, and termination of VF. On the one hand, elevated myocyte Ca^2+^ can cause delayed afterdepolarizations, triggered activity, and consequently life-threatening ventricular tachyarrhythmias in various pathological conditions such as digitalis toxicity, myocardial ischemia, or heart failure. On the other hand, VF itself directly and rapidly causes progressive myocyte Ca^2+^ overload that maintains VF and renders termination of VF increasingly difficult. Accordingly, energy levels for successful electrical defibrillation (defibrillation thresholds) increase as both VF and Ca^2+^ overload progress. Furthermore, VF-induced myocyte Ca^2+^ overload can promote re-induction of VF after defibrillation and/or postfibrillatory myocardial dysfunction (postresuscitation stunning) due to reduced myofilament Ca^2+^ responsiveness. The probability of these adverse events is best reduced by early detection and rapid termination of VF to prevent or limit Ca^2+^ overload. Early additional therapy targeting transsarcolemmal Ca^2+^ entry, particularly during the first 2 min of VF, may partially prevent myocyte Ca^2+^ overload and thus, increase the likelihood of successful defibrillation as well as prevent postfibrillatory myocardial dysfunction.

##  Myocyte Ca^2+^ overload initiates ventricular fibrillation

Elevated myocyte Ca^2+^ (Ca^2+^ overload) has generally been accepted to be responsible for the initiation of potentially lethal ventricular tachyarrhythmias including ventricular fibrillation (VF) in various pathological conditions such as digitalis toxicity, myocardial ischemia, or heart failure [1-4]. Specifically, the accumulation of Ca^2+^ in cardiomyocytes has long been suggested to cause delayed afterdepolarizations, triggered activity, and consequently life-threatening ventricular tachyarrhythmias [4,5]. Accordingly, myocyte Ca^2+^ overload has been shown to be related to the initiation of tachyarrhythmic activity in isolated hearts or in cardiomyocytes of rats or ferrets using bioluminescence or fluorescence of intracellular Ca^2+^ indicators (e.g. aequorin or indo-1) [1,6]. Further evidence of the importance of myocyte Ca^2+^ for the vulnerability to VF arises from a close correlation between myocyte Ca^2+^ levels and VF thresholds [2]. Controlled intracellular Ca^2+^ accumulation by programmed rapid ventricular stimulation (minimizing the effects of other arrhythmogenic factors) led to a parallel increase of VF thresholds in isolated rat hearts under nonischemic conditions [2]. 

In general, when Ca^2+^ loading of cardiomyocytes becomes sufficiently high, the sarcoplasmic reticulum can generate spontaneous Ca^2+^ oscillations that are not triggered by sarcolemmal depolarizations [1,3,8,6]. If sufficiently synchronized, these Ca^2+^ oscillations may cause delayed afterdepolarizations and initiate VF or modulate the initiation of VF [3]. Additionally, myocyte Ca^2+^ overload may facilitate the initiation of VF by Ca^2+^-induced cell-to-cell uncoupling [4], thereby slowing conduction and amplifying the tendency for reentrant arrhythmias. This tendency is particularly amplified in the hypertrophied heart where repolarization is heterogeneous and refractoriness prolonged [5]. Similarly, mutations in Ca^2+^ handling proteins have been suggested to contribute to hereditary arrhythmias. For example, defective ryanodine type 2 receptors (calcium release channels at the sarcoplasmic reticulum) or reduced levels of calsequestrin (a high-capacity Ca^2+^ binding protein expressed inside the sarcoplasmic reticulum), may cause increased Ca^2+^ discharge from the sarcoplasmic reticulum, and consequently ventricular tachyarrhythmias and sudden cardiac death induced by exercise, stress, or heart failure [7-9].

## VF causes myocyte Ca^2+^ overload

**Figure 1:**
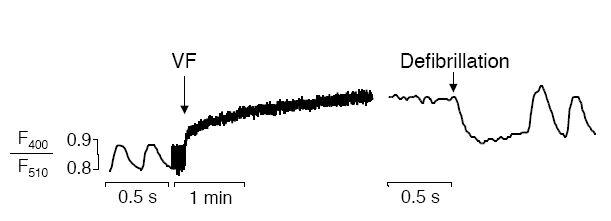
Original tracings of indo-1 fluorescence ratio transients (F400/F510), an index of myocyte Ca^2+^, in intact perfused rat hearts after the initiation of sustained VF (induced by 1-min rapid pacing at 20 Hz) and after electrical defibrillation. Note that myocyte Ca^2+^ rises rapidly and steeply upon VF to decrease again upon defibrillation.

Furthermore, myocyte Ca^2+^ and VF are mutually related. Myocyte Ca^2+^ overload can induce VF and conversely, VF itself causes myocyte Ca^2+^ overload [2,10-13]. Importantly, our studies using the Ca^2+^ sensitive fluorescent dye indo-1 in isolated rat hearts suggest that myocyte Ca^2+^ rises biphasically during VF (Figure 1). In the first 2 min of VF, mean myocyte Ca^2+^ rises steeply and rapidly reaches about double of normal levels. Thereafter, myocyte Ca^2+^ continues to rise but at a slower rate [11-14]. Additionally, successful defibrillation (electrical or pharmacological) led to a sudden reduction of VF-induced myocyte Ca^2+^ overload (Figure 1) [10]. In contrast, failed defibrillation shocks did not alter Ca^2+^ [10]. This demonstrates that VF directly and (dependent on VF duration) reversibly causes myocyte Ca^2+^ overload. Because the Ca^2+^ channel blocker diltiazem (1 μM) largely prevented VF-induced myocyte Ca^2+^ overload in the initial phase of VF in our experiments, most of the Ca^2+^contributing to myocyte Ca^2+^ overload presumably enters the cells through L-type Ca^2+^ channels [15]. This is likely due to the rapid activation rate in VF as the pacing cycle length was inversely related to both the rate and the degree of myocyte Ca^2+^overload induced by rapid pacing [2]. As VF persists, the contribution of Ca^2+^ entry through L-type Ca^2+^ channels to myocyte Ca^2+^ overload appears to decrease because diltiazem perfusion after 5 min of VF could not prevent myocyte Ca^2+^ to increase further in perfused rat hearts [10]. At this stage, further myocyte Ca^2+^ overload may arise from sarcoplasmic reticular Ca^2+^ release, from reverse Na+/Ca^2+^ exchange and/or from other sources (whereas individual contributions may vary species-dependently).

## VF-induced myocyte Ca^2+^ overload maintains VF

**Figure 2:**
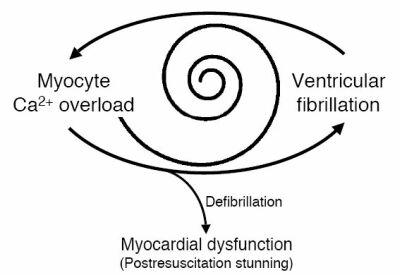
Myocyte Ca^2+^ overload and VF form a vicious circle in which elevated Ca^2+^ can induce VF and conversely, VF promotes Ca^2+^ overload maintaining the arrhythmia. As both VF and Ca^2+^ overload progress, energy levels for successful electrical defibrillation increase (symbolized by spiral). If defibrillation succeeds, VF-induced Ca^2+^ overload may cause postfibrillatory myocardial dysfunction (postresuscitation stunning).

Independent of the Ca^2+^ source, VF-induced myocyte Ca^2+^ overload contributes to maintain VF, leading to a self-maintaining vicious circle in which termination of VF becomes increasingly difficult (Figure 2). Consequently, myocyte Ca^2+^ overload can cause electrical defibrillation to fail and postshock re-induction of VF [10]. Accordingly, we could show that energy levels for successful electrical defibrillation (defibrillation thresholds) increase as both VF and Ca^2+^ overload progress [10]. Manipulating myocyte Ca^2+^ before defibrillation (increasing extracellular Ca^2+^ during VF in perfused rat hearts) we could demonstrate a causal relationship between myocyte Ca^2+^ concentrations and defibrillation success. Thus, the longer VF lasts, the higher both myocyte Ca^2+^ concentration and defibrillation threshold rise. This relationship was not due to prolonged myocardial ischemia because the hearts were continuously perfused during VF (normal levels of coronary flow, of coronary effluent pH, and of myocardial O2 consumption) [15]. Moreover, with increasing duration of VF, modulation of intracellular Ca^2+^ gets more difficult. Neither the Ca^2+^ channel blocker diltiazem (in a negative inotropic concentration of 1 μM) [16] nor low extracellular Ca^2+^ (reduction from 3.0 mM to 0.6 mM) could significantly decrease myocyte Ca^2+^ in fibrillating rat hearts. [10] Accordingly, diltiazem or low extracellular Ca^2+^ could not decrease defibrillation thresholds [10] as previously found for verapamil, another Ca^2+^ channel blocker, in pigs [17] or human beings in vivo [18]. The mechanism by which VF-induced myocyte Ca^2+^ overload increases defibrillation thresholds is probably related to a Ca^2+^-induced increase in the likelihood of defibrillation shocks to re-induce VF. We have previously shown that Ca^2+^ modulates the induction of VF by an electrical stimulus applied during the vulnerable period of repolarization [2,19]. As some portion of the fibrillating myocardium is always repolarizing [20], myocyte Ca^2+^ overload could increase the likelihood of a shock to re-induce VF. Thus, a shock applied to Ca^2+^ overloaded myocardium may terminate VF but simultaneously re-induce it by stimulating myocardium that is in the vulnerable period of repolarization. Furthermore, the chances for re-induction of VF increase as VF persists because normalization of  myocyte Ca^2+^ becomes increasingly difficult. Incomplete reduction of myocyte Ca^2+^ overload after initially successful defibrillation can be followed by synchronized spontaneous Ca^2+^ oscillations from the sarcoplasmic reticulum and subsequent reinduction of VF [10]. Because VF inevitably causes myocyte Ca^2+^ overload, this vicious circle between myocyte Ca^2+^ and VF might be a critical mechanism of failed defibrillation and postshock re-induction of VF. Moreover, this vicious circle concept suggests that the probability of these events is best reduced by early detection and rapid termination of VF to prevent or limit Ca^2+^ overload, and of course to prevent cerebral ischemia.

## Myocyte Ca^2+^ overload causes myocardial stunning after defibrillation (postresuscitation stunning)

Even if the self-maintaining vicious circle of Ca^2+^ and VF is interrupted and defibrillation succeeds, myocyte Ca^2+^ overload continues to cause problems. This is because transitory Ca^2+^ overload that occurs during VF can lead to reduced myofilament Ca^2+^ responsiveness [15] and consequently to postfibrillatory myocardial dysfunction [11] [15], a condition that we have termed postresuscitation stunning [15]. We found that the degree of Ca^2+^ overload during VF was inversely associated with the reduction of myofilament Ca^2+^ responsiveness after pacing-induced VF in our experiments in isolated rat hearts [11,15]. Accordingly, as Ca^2+^ overload progressed during VF, longer episodes of VF led to a more pronounced myocardial dysfunction than short episodes of VF. Moreover, increasing or decreasing Ca^2+^ overload during VF led to parallel changes in myofilament Ca^2+^ responsiveness (estimated as ratio of left ventricular developed pressure over myocyte Ca^2+^ transient amplitudes). The molecular mechanisms whereby transitory Ca^2+^ overload undermines contractile protein function seems to be related to proteolysis that is mediated at least partly by Ca^2+^-activated proteases (calpains) [21]. The substrates of calpains with respect to cardiac myofibrillar proteins include troponin I, troponin T, and others [21].

Postresuscitation stunning may be clinically relevant because defibrillation frequently results in a depressed myocardial function [22], particularly after prolonged VF [23]. Despite hemodynamic support, this dysfunction may be responsible for deaths after initially successful defibrillation [23]. In patients, however, VF is undoubtedly accompanied by myocardial ischemia. Therefore, global postischemic stunning most likely contributes to postfibrillatory myocardial dysfunction. Nevertheless, postfibrillatory myocardial dysfunction occurred even in the absence of ischemia or acidosis [11,15]. These results suggests that at least part of the postfibrillatory myocardial dysfunction is due to reduced myofilament Ca^2+^ responsiveness following VF-induced myocyte Ca^2+^ overload. Therefore, postfibrillatory myocardial dysfunction presumably is a consequence of reduced myofilament Ca^2+^ responsiveness following myocyte Ca^2+^ overload caused by both VF and consequent ischemia. Further contributions to postfibrillatory myocardial dysfunction may arise from ischemia-induced oxygen free radicals and/or from the impact of electrical defibrillation shocks. However, it seems that only high energy shocks of monophasic waveform may precipitate myocardial injury and dysfunction. Biphasic shocks of normal energy (up to 15 J/g wet heart weight, that is about 2-5 times the energy used in implantable defibrillator devices in relation to the myocardial mass) [15] appear not to significantly contribute to this dysfunction [15,24].

## Comparison to atrial fibrillation

The same vicious circle described above for myocyte Ca^2+^ and VF may also apply to atrial fibrillation (AF). It has been proposed that with increasing duration of paroxysmal AF, the likelihood that it will become chronic increases in parallel[25]. This so called "AF begets AF" concept seems to be due to *electrical remodeling* of the atria involving myocyte Ca^2+^ overload[26]. Atrial electrical remodeling seems also to be responsible for reinitiating AF after cardioversion because pretreatment with a Ca^2+^ channel blocker [26-28] or ryanodine (sarcoplasmic reticulum Ca^2+^ release blocker) [29] attenuated acute AF-induced electrophysiological changes [26-28] and reduced [27] or even eliminated [29]  re-initiation of AF. Furthermore, similar to postfibrillatory  ventricular dysfunction described above, depressed atrial contractile function persists after AF and cardioversion [30] even though atrial tissue is not (or only to a minor degree) ischemic during AF. Accordingly, atrial contractile dysfunction was reduced by verapamil but increased by the Ca^2+^ agonist BAY K8644, [30] suggesting that transsarcolemmal Ca^2+^ influx contributed to this dysfunction. All these observations fit in our vicious circle concept, whereby AF-induced Ca^2+^ overload may be responsible for the progressive nature of AF and for the re-initiation of AF as well as atrial contractile dysfunction after cardioversion.

## Species considerations

Extrapolation of the experimental findings in this article to the clinical setting should consider inter-species differences of excitation-contraction coupling. Most of the findings that underlie the proposed concept of Ca^2+^ and VF forming a vicious circle, arise from studies in cardiomyocytes or in isolated hearts of rats or ferrets. Importantly, the central role of Ca^2+^ in excitation-contraction coupling involving Ca^2+^-induced Ca^2+^ release in cardiac muscle physiology [31] suggests that VF leads to myocyte Ca^2+^ overload in most species including adult human beings. However, important inter-species and developmental differences exist regarding Ca^2+^-induced Ca^2+^ release from the sarcoplasmic reticulum and regarding Ca^2+^ removal processes [31]. For example, Ca^2+^- induced Ca^2+^ release was absent in frog or prenatal rat ventricle, intermediate in human ventricle, and most prominent in adult rat ventricular myocytes [31]. Consequently, activator Ca^2+^ in cardiac muscle of various species depends on different contributions from the sarcoplasmic reticulum, from L-type Ca^2+^ channels, and from forward Na+/Ca^2+^ exchange [31]. The kinetics and the degree of VF-induced myocyte Ca^2+^ overload may therefore vary among species and be part of the reason why some species are better protected against sustained VF than others (similar to myocardial mass). Based on the foregoing, VF in adult human beings most likely induces cardiomyocyte Ca^2+^ overload and the Ca^2+^ sources of this overload may be slightly but not fundamentally different from adult rat ventricles. Finally, similarities of myofilament Ca^2+^ sensitivity [31] and molecular mechanisms for myocardial stunning among species [21] suggest that VF-induced myocyte Ca^2+^ overload is an important and species-independent part of postfibrillatory myocardial stunning. This is why the proposed vicious circle of Ca^2+^ overload is likely to be of importance in human VF (and may be in AF too).

## Conclusions and clinical implications

Cardiomyocyte Ca^2+^ and VF are mutually related forming a self-maintaining vicious circle in the initiation, maintenance, and termination of VF. On the one hand, various experimental studies have shown that elevated myocyte Ca^2+^ can cause delayed afterdepolarizations, triggered activity, and consequently life-threatening ventricular tachyarrhythmias. On the other hand, VF itself directly and rapidly causes progressive myocyte Ca^2+^ overload that maintains VF and renders termination of VF increasingly difficult. Accordingly, VF-induced myocyte Ca^2+^ overload can cause electrical defibrillation to fail. Furthermore, VF-induced myocyte Ca^2+^ overload can promote re-induction of VF after defibrillation and/or postfibrillatory myocardial dysfunction (postresuscitation stunning) due to reduced myofilament Ca^2+^ responsiveness. The same vicious circle may apply to AF during electrical remodeling to cause re-initiation of AF and atrial contractile dysfunction after cardioversion. The experimental studies reviewed here suggest that the probability of these adverse events is best reduced by early detection and rapid termination of VF to prevent or limit Ca^2+^ overload. In other words, rapid termination of VF not only reduces the risk of cerebral injury but also the likelihood of failed defibrillation and postfibrillatory myocardial dysfunction. Early additional therapy targeting transsarcolemmal Ca^2+^ entry, particularly during first 2 min of VF, may partially prevent myocyte Ca^2+^ overload and thus, increase the likelihood of successful defibrillation as well as prevent postfibrillatory myocardial dysfunction. Thereafter, the vicious circle of myocyte Ca^2+^ overload is established and therapeutic attempts have an inherently lower likelihood of success.
